# A Functional Data Analysis Approach for Circadian Patterns of Activity of Teenage Girls

**DOI:** 10.5334/jcr.ac

**Published:** 2015-04-08

**Authors:** Ruzong Fan, Victoria Chen, Yunlong Xie, Lanlan Yin, Sungduk Kim, Paul S Albert, Bruce Simons-Morton

**Affiliations:** Biostatistics and Bioinformatics Branch, 6100 Executive Blvd, Room 7B07J, MSC 7510, Division of Intramural Population Health Research, Eunice Kennedy Shriver National Institute of Child Health and Human Development, National Institutes of Health, Bethesda, MD 20892; Glotech, 6100 Executive Blvd, Rockville, MD 20852; Health Behavior Branch, Division of Intramural Population Health Research, Eunice Kennedy Shriver National Institute of Child Health and Human Development, National Institutes of Health, Bethesda, MD 20892

**Keywords:** circadian rhythms, obesity, body mass index, functional data analysis

## Abstract

**Background:** Longitudinal or time-dependent activity data are useful to characterize the circadian activity patterns and to identify physical activity differences among multiple samples. Statistical methods designed to analyze multiple activity sample data are desired, and related software is needed to perform data analysis.

**Methods:** This paper introduces a functional data analysis (fda) approach to perform a functional analysis of variance (fANOVA) for longitudinal circadian activity count data and to investigate the association of covariates such as weight or body mass index (BMI) on physical activity. For multiple age group adolescent school girls, the fANOVA approach is developed to study and to characterize activity patterns. The fANOVA is applied to analyze the physical activity data of three grade adolescent girls (i.e., grades 10, 11, and 12) from the NEXT Generation Health Study 2009–2013. To test if there are activity differences among girls of the three grades, a functional version of the univariate *F*-statistic is used to analyze the data. To investigate if there is a longitudinal (or time-dependent activity count) difference between two samples, functional *t*-tests are utilized to test: (1) activity differences between grade pairs; (2) activity differences between low-BMI girls and high-BMI girls of the NEXT study.

**Results:** Statistically significant differences existed among the physical activity patterns for adolescent school girls in different grades. Girls in grade 10 tended to be less active than girls in grades 11 & 12 between 5:30 and 9:30. Significant differences in physical activity were detected between low-BMI and high-BMI groups from 8:00 to 11:30 for grade 10 girls, and low-BMI group girls in grade 10 tended to be more active.

**Conclusions:** The fda approach is useful in characterizing time-dependent patterns of actigraphy data. For two-sample data defined by weight or BMI values, fda can identify differences between the two time-dependent samples of activity data. Similarly, fda can identify differences among multiple physical activity time-dependent datasets. These analyses can be performed readily using the fda R program.

## 1 Introduction

Longitudinal or time-dependent activity data are useful to characterize the physical activity patterns and to identify activity differences among multiple samples. Statistical methods designed to analyze activity data collected annually are desired, and related software is needed to perform routine data analysis. In particular, the methods which may characterize the temporal trends and differences of the activity data are important and needed[8]. It is a tradition in the circadian research to employ simple cosinor models or harmonic modeling approaches to detect the 24-hour activity patterns in the activity data and to compare amplitude and phase shifts between groups of interest[7,9,10]. However, analyses of these data could benefit from functional-based approaches.

In this paper, we develop a functional data analysis (fda) approach to measure and analyze physical activity patterns in adolescent girls[5]. The functional data analysis focuses on an overall comparison of the two curves with temporal or point-wise (at multiple time points during the 24 hours) comparisons between groups. An advantage of the functional data analysis approach is that since all the tests are permutation-based, it is less sensitive to distributional assumptions. One may perform a functional analysis of variance (fANOVA) to detect the temporal activity difference of two or multiple samples. As examples, fANOVA methods are used to explore: (1) the role of body mass index (BMI) on the activity patterns among teenage girls over time; and (2) the differences in the activity patterns of teenage girls over consecutive years. As a second research objective, we investigate the variability by race and family affluence in activity for the adolescent girls.

Specifically, we utilize and analyze the physical activity data of adolescent girls in grades 10, 11, and 12 from the NEXT Generation Health Study 2009–2013. The NEXT Generation Health Study is a longitudinal study investigating the health behaviors of adolescents. The study contains a nationally-representative sample of U.S. students followed from grades 10–12. The goals of the NEXT study include (1) to identify the trajectory of adolescent health status and health behaviors from mid-adolescence through the post high school year; (2) to examine individual predictors of the onset of key adolescent risk behaviors and risk indicators during this period; (3) to identify family, school, and social/environmental factors that promote or sustain positive health behaviors; and (4) to identify transition points in health risk and risk behaviors and changes in family, school, and social/environmental precursors to these transitions.

The remainder of this article is organized as follows. In Section 2, we provide an introduction of the physical activity data of the NEXT study and a brief outline of the fANOVA methods we need. In Section 3, we show the analysis results of the NEXT study. Section 4 includes the discussion of the findings and some remarks.

## 2 Methods and Materials

### 2.1 Data

In the NEXT Generation Health Study, activity counts were measured using Actiwatch2 devices manufactured by Respironics Inc. (http://www.actigraphcorp.com/company/). In this study, we analyze physical activity data of adolescent girls of grades 10, 11, and 12 collected over 3 consecutive years using Actiwatches, respectively. Only female student data are used for our analysis. The demographic information of subjects in the three grades is provided in Table 1. In grade 10, the data of 95 students are available, and for grades 11 and 12, the activity data of 85 and 84 students are available due to some dropouts, respectively. Race/ethnicity includes four categories: “Hispanic”, “African American”, “Asian”, and “White”. The number of “Asian” students is very small (2 in grade 10, 1 in grade 11, and 2 in grade 12), and the numbers of the other three categories range from 23 to 35 and vary slightly among the three grades.

**Table 1:** Demographic information of subjects in Grades 10, 11, and 12.

Grade	Sample Size	Covariates		n (%) or Mean (SD)

10	N = 95	Race *	1: Hispanic	35 (36.84%)
			2: African American	25 (26.32%)
			3: Asian	2 (2.11%)
			4: White	33 (34.74%)
		BMI Group *	0: Low BMI Group	76 (80.00%)
			1: High BMI Group	19 (20.00%)
			BMI **	25.50 (5.44)
		Family Affluence *	1: Low Affluence	30 (31.58%)
			2: Moderate Affluence	43 (45.26%)
			3: High Affluence	22 (23.16%)
		Parental Education **	Parental Education 1	3.49 (1.92); n = 91
			Parental Education 2	3.13 (1.84); n = 60
11	N = 85	Race *	1: Hispanic	30 (35.29%)
			2: African American	25 (29.41%)
			3: Asian	1 (1.18%)
			4: White	29 (34.12%)
		BMI Group *	0: Low BMI Group	68 (80.00%)
			1: High BMI Group	17 (20.00%)
			BMI **	25.39 (5.32)
		Family Affluence *	1: Low Affluence	26 (30.59%)
			2: Moderate Affluence	38 (44.71%)
			3: High Affluence	21 (24.71%)
		Parental Education **	Parental Education 1	3.53 (1.98); n = 81
			Parental Education 2	3.21 (1.84); n = 53
12	N = 84	Race *	1: Hispanic	29 (34.52%)
			2: African American	23 (27.38%)
			3: Asian	2 (2.38%)
			4: White	30 (35.71%)
		BMI Group *	0: Low BMI Group	68 (80.95%)
			1: High BMI Group	16 (19.05%)
			BMI **	25.15 (5.38)
		Family Affluence *	1: Low Affluence	26 (30.95%)
			2: Moderate Affluence	39 (46.43%)
			3: High Affluence	19 (22.62%)
		Parental Education **	Parental Education 1	3.53 (1.92); n = 80
			Parental Education 2	3.09 (1.84); n = 53

* n (%) is the number (percentage) for each level of categorical covariate. ** Mean (SD) is the mean (standard deviation) of each continuous covariate. Parental Education 1 and 2 describe the highest level of education completed by each respective guardian in the household; education level is reported using a seven point scale.

The study data analyzed is a subsample of the NEXT data that oversampled overweight children. This subsample of 550 adolescents (NEXT-Plus Cohort) consisted of 50% overweight individuals. Two BMI groups, “Low BMI Group” vs. “High BMI Group”, are defined via pre-assigned categories based on percentiles of 550 adolescents: “Low BMI Group” was defined by < 95th percentile and ”High BMI Group” was defined by >= 95th percentile. This corresponds to the upper and lower 20% percentiles of the study data in this paper (Table 1). Family Affluence includes three categories: “Low Affluence”, “Moderate Affluence”, and “High Affluence”. Parental Education 1 and 2 describe the highest level of education completed by each respective guardian in the household; education level is reported using a seven point scale.

Physical activity counts were measured every 30 seconds using the Actiwatch2 devices. The Actiwatch activity monitor contains an omni-directional sensor sensitive to 0.01gravity (0.098 mzs22), and is capable of detecting acceleration in two planes. The sensor integrates the degree and speed of motion and produces an electrical current that varies in magnitude such that an increase in speed and motion produces an increase in voltage stored as activity counts. For this study, activity monitors were placed on participants’ wrists. Times in different time zones were accounted for to make sure that the time interval of physical activity counts started at 0:00 for each individual. Only full weekday 24-hour records from 0:00 – 23:59.5 with activity count sums greater than 30,000 are included. To make the activity data comparable, activity counts were collected in 30-second epoches for 7 consecutive days. To facilitate data analysis, the activity counts were summed up over every 15 minutes to give 4 observations an hour. In total, each student has *N* = 4 × 24 = 96 counts each day for the 7 consecutive days. Thus, each student has an accumulated activity count at each time point *t_i_* each day, where *t_i_* = *i/* 4, *i* = 0,1, 2, …, *N –* 1 = 95. Usable data are averaged over the 7 consecutive days at each time point for one full day, and so the final data consist of *N* = 96 observations for each individual.

### 2.2 Functional Data Analysis

Consider a sample with *n* subjects listed as individuals *i* = 1, *…, n.* For an individual *i*, assume that *N* activity counts are available at times *t*_1_, *…, t_N_.* The activity count at *t_j_* is denoted by *y_i_*(*t_j_*), *j*=1, *…, N*, and so the activity counts of individual *i* can be summarized as *Y_i_* = (*y_i_*(*t*_1_), *…, y_i_*(*t_N_*))[1]. The activity profile *y_i_*(*t*) of individual *i* is a function of time *t*, which can be estimated by *Y_i_.* To estimate the activity function *y_i_*(*t*) from the activity counts *Y_i_*, we use an ordinary linear square smoother[1,2,3,4,5]. Specifically, let φ*_k_*(*t*), *k* = 1, *…, K*, be a series of *K* basis functions, such as B-spline basis and Fourier basis functions. Let Φ denote the *N* by *K* matrix containing the values φ*_k_* (*t_j_*), where *j* ε 1, *…, N.* Using the discrete realizations *Y_i_* = (*y_i_*(*t*_1_), *…, y_i_*(*t_N_*))′, we may estimate the activity function *y_i_*(*t*) using an ordinary linear square smoother as follows (Ramsay and Silverman 2005, Chapter 4)[4]

1 \[ {\hat y_i}(t) = ({y_i}({t_1}), \cdots ,{y_i}({t_N}))\Phi {[\Phi
                '\Phi ]^{ - 1}}\phi (t), \]

where φ(*t*) = (φ_1_(*t*), *…*, φ*_K_*(*t*))′. The estimate *ŷ_i_*(*t*) smoothes activity patterns over time. In this article, we consider the Fourier basis functions: φ_0_(*t*) = 1, φ_2_*_r-_*_1_(*t*) = sin(2π*rt/N*), and φ_2_*_r_*(*t*) = cos(2π*rt/N*), *r* = 1, *…*, (*K –* 1)*/*2, where *K* is taken as a positive odd integer. In our analysis of the physical activity data of NEXT study, *K* is taken a value of 25. We also try other values such as *K* = 21, 23, and 27, and the results are similar to those of *K* = 25. One may use B-spline basis functions, but the activity data are likely to be periodic and Fourier basis functions make more sense[1,2,3,4,5].

We are interested in whether there are activity differences between two BMI groups and between grade pairs for the adolescent girls at every time point. To investigate differences between two groups, we use permutation *t*-tests. Let [*x*_11_(*t*), *…, x*_1_*_n_*_1_(*t*)] and [*x*_21_(*t*), *…, x*_2_*_n_*_2_] be two sub-samples of activity functions with sample sizes *n*_1_ and *n*_2_. For each time value *t*, we consider the absolute value of a *t*-statistic to evaluate the difference

2 \[ T(t) = \frac{{|{{\bar x}_1}(t) - {{\bar x}_2}(t)|}}{{\sqrt
                {Var[{x_1}(t)]/{n_1} + Var[{x_2}(t)]/{n_2}} }}, \]

where \[{\bar x_1}(t)\] and \[{\bar x_2}(t)\] are sample mean functions of [*x*_11_(*t*), *…, x*_1_*_n_*_1_(*t*)] and [*x*_21_(*t*), *…, x*_2_*_n_*_2_], and Var[*x*_1_(*t*)] and Var[*x*_2_(*t*)] are variance functions of functions of *x*_1_*_i_*(*t*) and *x*_2_*_i_*(*t*), respectively.

To test if there are activity differences among the three grade adolescent girls, we use a functional version of the univariate *F*-statistic. Let *y_i_*(*t*), *i* = 1, …, *n*, be a sample consisting of three sub-samples of activity count functions of grades 10, 11, and 12. In addition, let *x_ij_* take value 1 or 0 which indicates if the activity count function is from *j* = 1 for grade 10, *j* = 2 for grade 11, and *j* = 3 for grade 12. For instance, *x_i_*_1_ = 1 indicates that *y_i_*(*t*) is from grade 10, and *x_i_*_1_ = 0 indicates that *y_i_*(*t*) is from grade 11 or 12. One may want to notice that one and only one of *x_i_*_1_, *x_i_*_2_, and *x_i_*_3_ is equal to 1, and so the summation *x_i_*_1_ + *x_i_*_2_ + *x_i_*_3_ is equal to 1 for all *i*. Consider the following functional linear model of functional activity data

3 \[ {y_i}(t) = {\beta _0}(t) + \mathop \sum \limits_{j = 1}^3
                {x_{ij}}{\beta _j}(t) + {\varepsilon _i}(t), \]

where β_0_(*t*) is functional intercept, β*_j_*(*t*) is functional regression coefficient of *x_ij_*, and ε*_i_*(*t*) is the functional error term. The functional version of the univariate *F*-statistic is defined by

4 \[ F(t) = \frac{{Var[\hat y(t)]}}{{\sum {{[{y_i}(t) - \hat
                y(t)]}^2}/n}}, \]

where *ŷ*(*t*) are the predicted values from the functional linear model (3)[5].

To find a critical value of this statistic, we use a permutation test. We perform the following procedure: (1) randomly shuffle the labels of the smoothed activity functions; (2) recalculate the maximum of *T*(*t*) or *F*(*t*) with the new labels. Repeating this many times allows a null distribution of no activity difference to be constructed. This provides a reference for evaluating the maximum value of the observed *T*(*t*) or *F*(*t*). In our analysis, we execute a permutation test *T*(*t*) or *F*(*t*) by a default value of 200 random shuffles. A *p*-value of the test *T*(*t*) or *F*(*t*) is the proportion of permutation *T*(*t*) or *F*(*t*) values that the maximum of *T*(*t*) or *F*(*t*) are larger than the *T_Obs_*(*t*) or *F_Obs_*(*t*) statistics for the observed one. As suggested in Ramsay JO, Hooker G, and Graves S, two different ways are used to calculate the *p*-values: global test and point-wise test. The global test provides a single *p*-value level which is the proportion of maximized *T*(*t*) or *F*(*t*) values that are larger than maximized *T_Obs_*(*t*) or *F_Obs_*(*t*) at all time points *t*. The point-wise test provides a curve which is the proportion of all permutation *T*(*t*) or *F*(*t*) values which are larger than the observed *T_Obs_*(*t*) or *F_Obs_*(*t*) at each time point *t*.

## 3 Results

**Figure 1:**
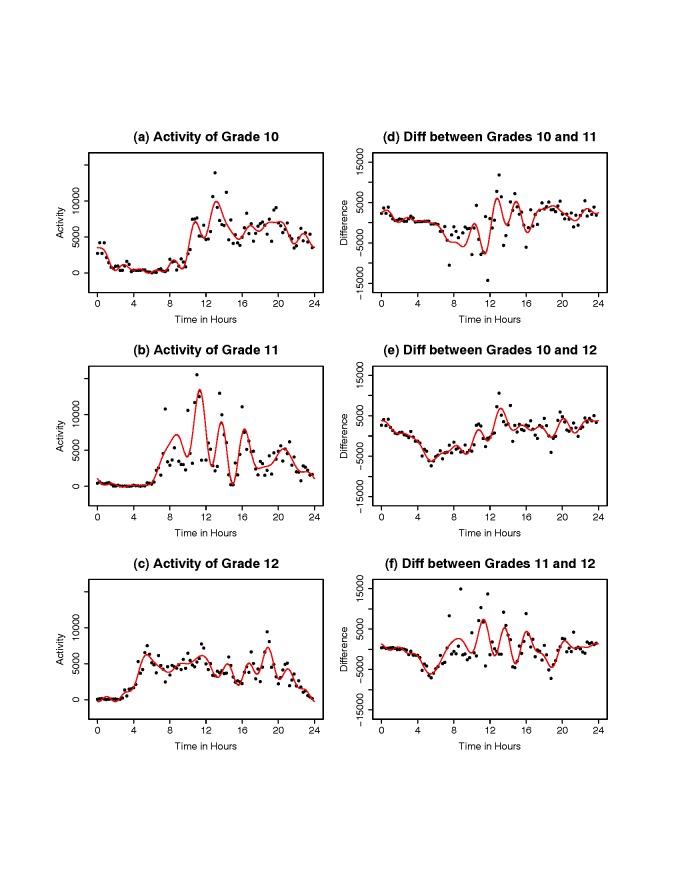
**Activity patterns and activity differences of subject 909010214**. In the left-hand side plots (a), (b), and (c), activity counts are shown by black dots. In the right-hand side plots (d), (e), and (f), the differences of activity counts between the three pairs of grades are shown by black dots. The smoothed Fourier expansion in each plot is shown by a red solid curve. Abbreviation: Diff = Difference.

**Data display and smoothed activity functions.** We smoothed each individual’s activity counts by the linear square smoother defined in relation (1). Figure 1 shows activity patterns and activity differences by grade of subject 909010214. In the Figure, activity counts over time are shown by black dots on the left-hand side plots (a), (b), and (c). In the right-hand side plots (d), (e), and (f), the difference of activity counts between three pairs of grades are shown by black dots. The smoothed Fourier expansion in each plot is shown by the red solid curve. Figure 1 suggests that there are activity differences among the three grade girls since the differences are not always around 0 in the daytime.

**Figure 2:**
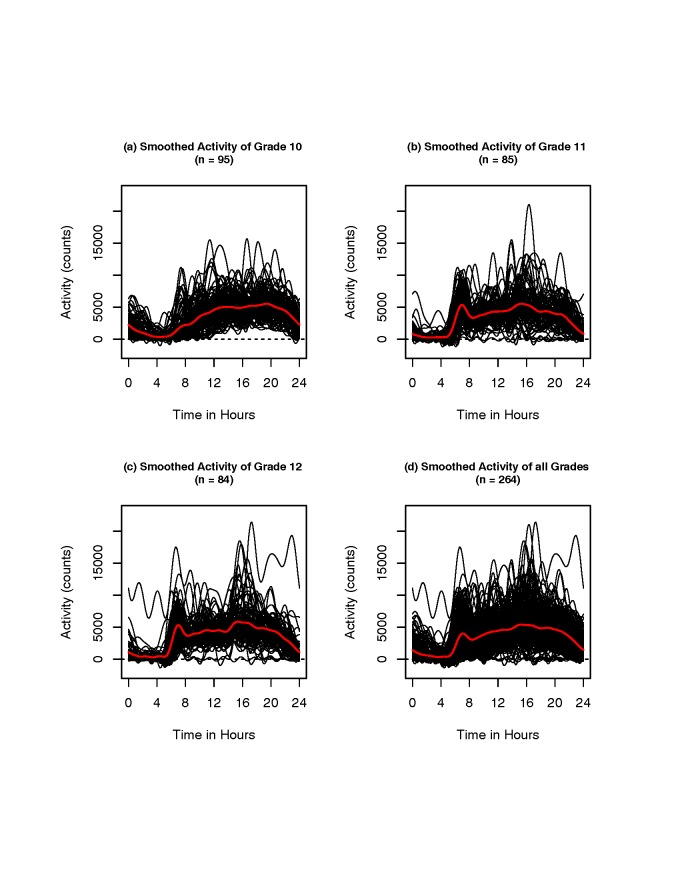
**Smoothed activity patterns**. In the plots (a), (b), and (c), the individual smoothed Fourier expansions of grades 10, 11, and 12 are shown respectively. In plot (d), smoothed Fourier expansions of all three grades are shown.

**Smoothed activity patterns and difference among grades.** Figure 2 shows smoothed activity patterns of girls by grade and combinations of all activity data. In the plots (a), (b), and (c), the individual smoothed Fourier expansions of activity data of grades 10, 11, and 12 are shown, respectively. In plot (d), smoothed Fourier expansions of combinations of all activity data of three grade girls are shown. Each individual’s smoothed activity pattern is represented by a black line. The mean activity pattern across all subjects is shown by the red line in each plot. In the plots (b) and (c), there is an activity peak between 5:30 and 9:30 for grades 11 and 12 girls, but no peak for the grade 10 girls in plot (a) in the time interval.

**Figure 3:**
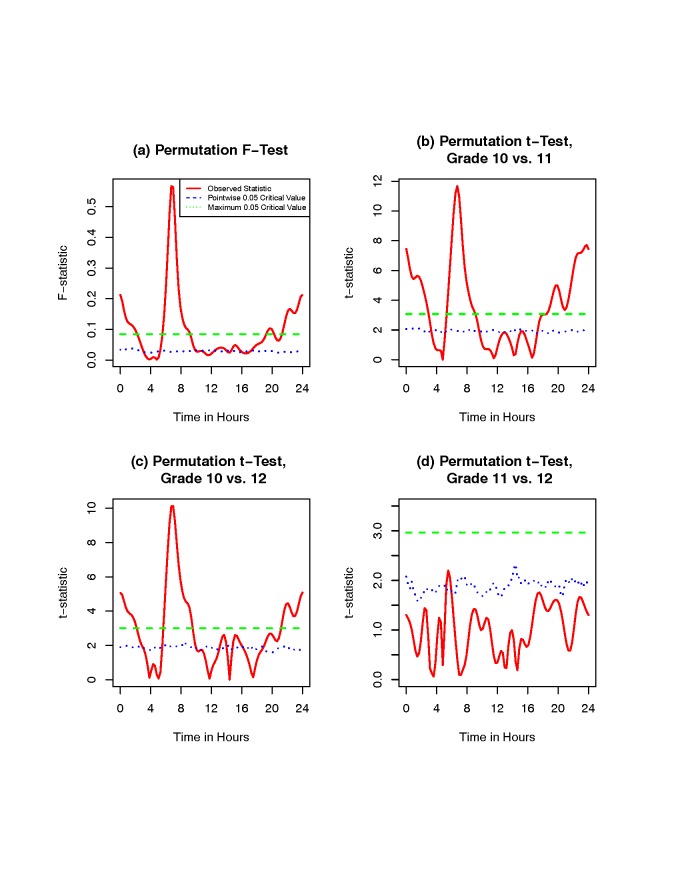
**Observed *F*-statistic and *t*-statistic results and related permutation critical values for a relationship between activity counts and grades.** The red solid curve represents the observed statistic *F*(*t*) or *T*(*t*) at each time point, and the green dashed and blue dotted lines correspond to permutation critical values for the maximum and point-wise statistics at significance level α = 0.05, respectively.

Figure 3 shows the observed *F*-statistic and *t*-statistic results and related permutation critical values for a relationship between activity counts and grade girls. The red solid curve represents the observed statistic *F*(*t*) or *T*(*t*) at each time point, and the green dashed and blue dotted lines correspond to permutation critical values for the maximum and point-wise statistics at a significance level α = 0.05, respectively. When *F*(*t*) or *T*(*t*) is above the green dashed or blue dotted line, the two/three grade girls have significantly different mean activity patterns at those time points. The global critical value (green dashed line) is preferred since it is more conservative. Thus, we use the global critical value to check if there are activity differences in a time interval.

The plot (a) in Figure 3 shows that there are activity differences among the three grade girls in two time intervals: one from 5:30 to 9:30, and the other from 22:00 to 2:00. The plots (b) and (c) of Figure 3 show that the physical activity pattern in girls of grade 10 is significantly different from the patterns observed in girls of grades 11 and 12 in these two time intervals. The plot (d) of Figure 3 reveals that the physical activity patterns in girls of grades 11 and 12 are not significantly different from each other. Combining the results of Figures 2 and 3, the grade 10 girls have lower activity than girls of grades 11 and 12 between 5:30 and 9:30. Therefore, physical activity patterns in grade 10 girls differ significantly from the activity patterns in girls of both grades 11 and 12, which is consistent with the smoothed activity patterns in Figure 2.

**Figure 4:**
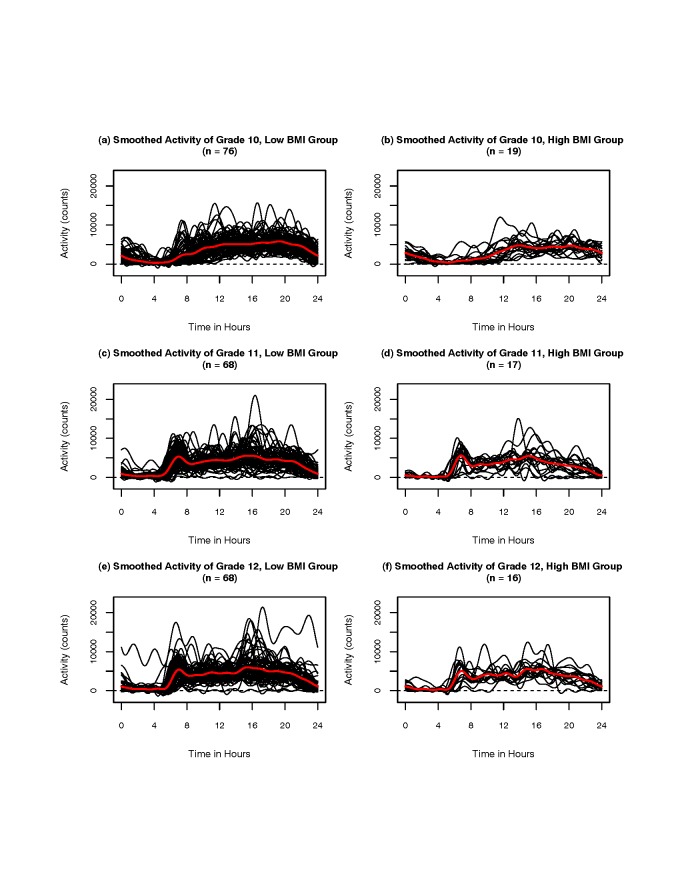
**Smoothed activity patterns by BMI status in the three grades**. In the plots (a) and (b), the individual smoothed Fourier expansions of grade 10 are shown for low-BMI vs. high-BMI groups, respectively. In the plots (c) and (d), the individual smoothed Fourier expansions of grade 11 are shown for low-BMI vs. high-BMI groups, respectively. In the plots (e) and (f), the individual smoothed Fourier expansions of grade 12 are shown for low-BMI vs. high-BMI groups, respectively.

**Figure 5:**
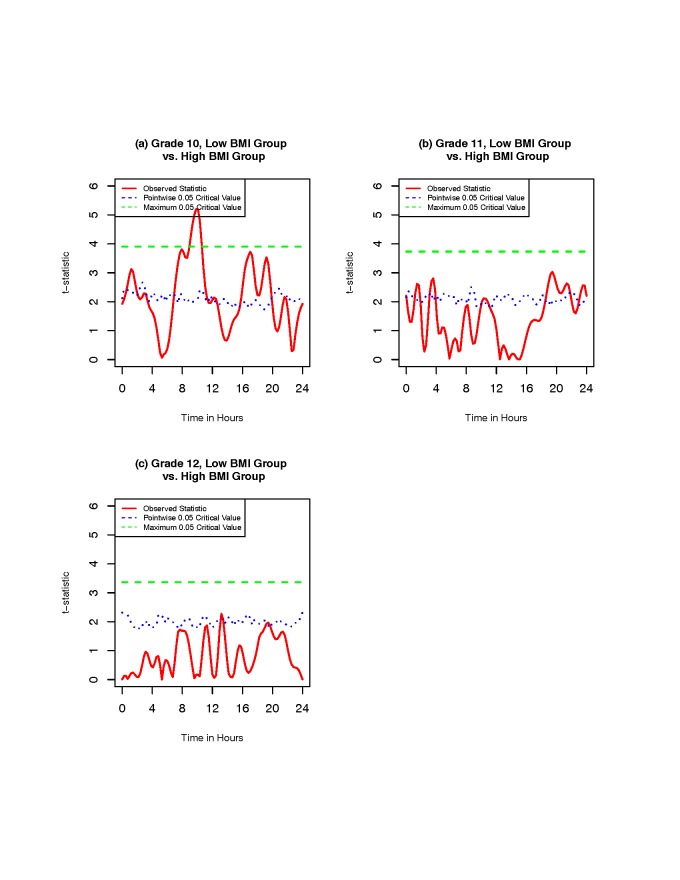
**Observed *t*-statistic results and related permutation critical values for the activity counts between low-BMI and high-BMI groups in the three grades.** The red solid curve represents the observed statistic *T*(*t*) at each time point, and the green dashed and blue dotted lines correspond to permutation critical values for the maximum and point-wise *t*-statistics at significance level α = 0.05, respectively.

**Smoothed activity patterns and difference between BMI groups**. Figure 4 shows the smoothed activity patterns by BMI status in the three grades. In the plots (a) and (b), the individual smoothed Fourier expansions of grade 10 girls are shown for low-BMI vs. high-BMI groups, respectively. In the plots (c) and (d), the individual smoothed Fourier expansions of grade 11 girls are shown for low-BMI vs. high-BMI groups, respectively. In the plots (e) and (f), the individual smoothed Fourier expansions of grade 12 girls are shown for low-BMI vs. high-BMI groups, respectively. From the plots (a) and (b) of grade 10 girls, the smoothed activity functions of low-BMI group are generally higher than those of high-BMI group. Figure 5 shows the observed *t*-statistic results and related permutation critical values for the activity counts between low-BMI and high-BMI groups in the three grades. There is a significant activity difference between the low-BMI and high-BMI groups in grade 10 girls [plot (a) in Figure 5], but not in girls of grades 11 and 12 [plots (b) and (c) in Figure 5]. The difference in grade 10 girls occurs between 8:00 and 11:30. Ogbagaber et al. (2014)[7] found that there is a significant difference between the low-BMI and high-BMI groups in grade 10 girls, and the result in plot (a) of Figure 5 confirms the result.

**Figure S.1:**
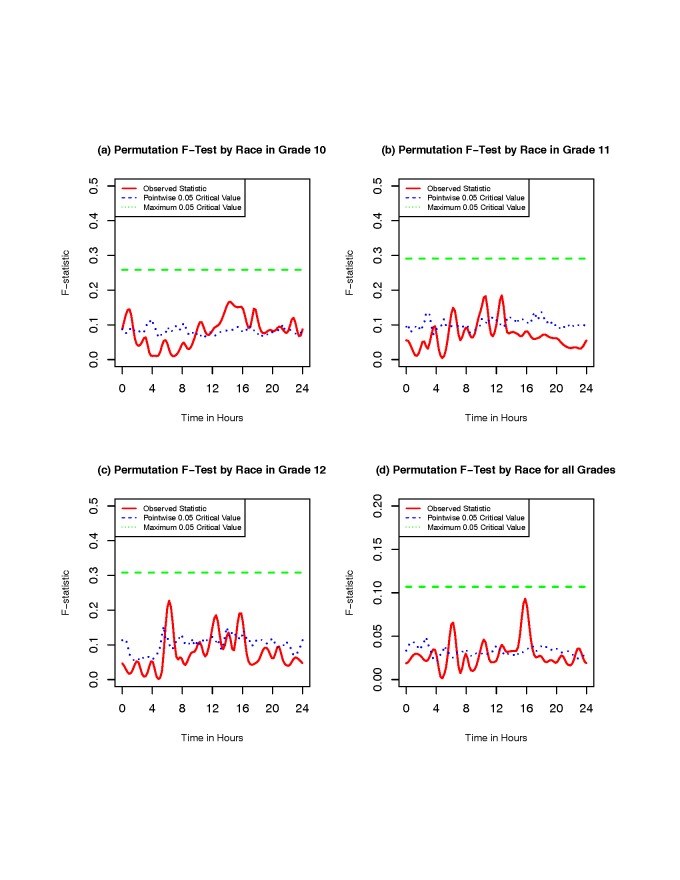
**Observed *F*-statistic results and related permutation critical values for a relationship between activity counts and race/ethnicity.** The red solid curve represents the observed statistic *F*(*t*) at each time point, and the green dashed and blue dotted lines correspond to permutation critical values for the maximum and point-wise statistics at significance level α = 0.05, respectively.

**Activity differences among three categories of race/ethnicity and among family affluence**. In our analysis, we investigated if there are activity differences among three categories of race/ethnicity: “Hispanic”, “African American”, and “Asian and White” (i.e., “Asian” students are combined into a single category with ”White” students due to the small number of Asians). In addition, we examined activity differences among the three categories of family affluence. No significant differences were found for the race/ethnicity or family affluence categories since the observed *F*-statistic is below the green dashed critical values in Figures S.1 and S.2 as displayed in the **Supplementary Materials**.

## 4 Discussion

This paper introduced a functional data analysis approach to perform an fANOVA for physical activity count data and to investigate the impact of covariates such as weight or BMI on physical activity. The fANOVA approach was developed to study changes in circadian rhythms across longitudinal follow-ups, i.e., to study and to characterize the activity patterns of physical activity across multiple grade girls. The fANOVA was applied to analyze the ­physical activity data of three grade girls in the NEXT Generation Health Study 2009–2013. To test if there are activity differences among the three grade girls, a functional version of the univariate *F*-statistic was used to analyze the data. In addition, we examined activity differences among three categories of race/ethnicity or among the three categories of family affluence. To investigate if there is a temporal difference between two samples, functional *t*-tests were utilized to test the following: (1) activity differences between activity data of grade pairs; and (2) activity differences between low-BMI girls and high-BMI girls of the NEXT study. To get critical values of the tests, one may perform permutation tests to avoid problems of normal distribution assumptions of the count data.

Statistically significant differences existed among the activity patterns over time for adolescent school girls. The tenth grade girls tended to be less active than high grade adolescent school girls of grades 11 & 12 between 5:30 and 9:30 and between 22:00 and 2:00. Significant differences of physical activity were detected between low-BMI and high-BMI groups from 8:00 to 11:30 for grade 10 girls, and low-BMI group girls of grade 10 tended to be more active. For the school girls of grades 11 or 12, no significant difference existed in the physical activity patterns between low-BMI and high-BMI groups. No significant differences were found for race/ethnicity or family affluence categories.

The difference over time may be due to the way subjects were accrued. The original 95 participants were girls measured during the summer months (Tables S.1 and S.2 in the **Supplementary Materials**). In subsequent grades, some girls were measured in the spring or winter and were likely in school, working, or engaged in different activities than in the 10th grade. Also, 11th- and 12th-grade girls may have had more independence and less structure in daily activities relative to girls in the 10th grade. These may be reasons that the tenth grade girls tended to be less active. Activity tends to decline during high school as reported in Allison et al. (2007)[10].

The functional data analysis approach was proposed and used in Wang et al. (2011)[8] to analyze the physical activity data of two groups. The models and methods of this paper are similar to those of Wang et al. (2011)[8]. In our analysis, we analyzed physical activity data of two groups and three groups and the methods can be used to analyze data of higher numbers of groups. Ogbagaber et al. (2012)[7] developed a harmonic shape invariant model to estimate circadian cycles and to analyze the grade 10 data of the NEXT study. The fda approach is flexible since the R package fda can facilitate the implementation of the proposed methods easily. In addition, it is possible to adjust for covariates, e.g., the covariates can be added to the functional linear model (3).

We address the first three goals of the NEXT study outlined in the section of **Introduction** by analyzing physical activity data by functional data analysis approaches: (1) to study the trajectory of physical activity of adolescent girls; (2) to examine the effect of risk indicators such as BMI; and (3) to examine the effect of family affluence and race/­ethnicity on physical activity data. The analytic approaches presented in this paper are very general and can be applied to similar problems, such as physical activity data of both males and females in addition to BMI status. In this NEXT study, we only have female adolescent girls. In other studies, multiple sub-sample data may be available, and the methods from this paper can be very useful for future analyses.

## Figures and Tables

**Figure S.2:**
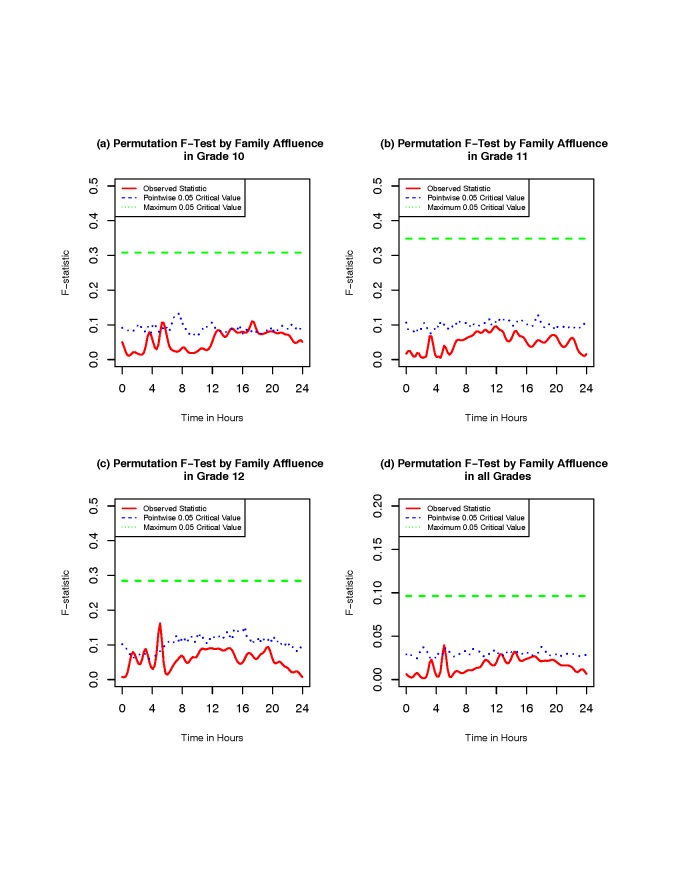
**Observed *F*-statistic results and related permutation critical values for a relationship between activity counts and family affluence.** The red solid curve represents the observed statistic *F*(*t*) at each time point, and the green dashed and blue dotted lines correspond to permutation critical values for the maximum and point-wise statistics at significance level α = 0.05, respectively.

## Supplementary Materials for A Functional Data Analysis Approach for Circadian Patterns of Activity of Teenage Girls

### Appendix A. Activity Measurement Recording Counts by Months

In the Table S.1, we provide the sub-sample sizes in grades 10, 11, and 12 by recording months. In the Table S.2, we provide the sub-sample sizes in grades 10, 11, and 12 by summer recording (i.e., months 6, 7, 8) vs. non-summer recording (i.e., months 1–5, 9–12).

**Table S.1:** Sub-sample Sizes in Grades 10, 11, and 12 by Recording Months.

Recording Month	Sub-sample Sizes		

	**Grade 10**	**Grade 11**	**Grade 12**

2	0	13	22
3	0	18	31
4	0	33	16
5	0	10	15
6	31	9	0
7	54	2	0
8	10	0	0
**Total**	95	85	84

**Table S.2:** Sub-sample Sizes in Grades 10, 11, and 12 by Summer Recording (i.e., Months 6, 7, 8) vs. Non-summer Recording (i.e., Months 1–5, 9–12).

Summer vs. Non-Summer	Sub-sample Sizes		

	**Grade 10**	**Grade 11**	**Grade 12**

**Summer**	95	11	0
**Non-summer**	0	74	84
**Total**	95	85	84

### Appendix B. Activity Measurement Recording Counts by Months And BMI Groups

In the Table S.3, we provide the sub-sample sizes in grades 10, 11, and 12 by recording months and BMI groups. In the Table S.4, we provide the sub-sample sizes in grades 10, 11, and 12 by summer recording (i.e., months 6, 7, 8) vs. non-summer recording (i.e., months 1–5, 9–12), and BMI groups.

**Table S.3:** Sub-sample Sizes in Grades 10, 11, and 12 by Recording Months, and Low BMI and High BMI Groups.

The Recording Month	Sub-sample Sizes					

	**Grade 10**		**Grade 11**		**Grade 12**	

	**Low BMI**	**High BMI**	**Low BMI**	**High BMI**	**Low BMI**	**High BMI**

2	0	0	2	11	5	17
3	0	0	3	15	8	23
4	0	0	7	26	3	13
5	0	0	3	7	0	15
6	4	27	2	7	0	0
7	13	41	0	2	0	0
8	2	8	0	0	0	0
**Total**	19	76	17	68	16	68

**Table S.4:** Sub-sample Sizes in Grades 10, 11, and 12 by Summer Recording (i.e., Months 6, 7, 8) vs. Non-summer Recording (i.e., Months 1–5, 9–12), and Low BMI and High BMI Groups.

The Recording Month	Sub-sample Sizes					

	**Grade 10**		**Grade 11**		**Grade 12**	

	**Low BMI**	**High BMI**	**Low BMI**	**High BMI**	**Low BMI**	**High BMI**

**Summer**	19	76	2	9	0	0
**Non-summer**	0	0	15	59	16	68
**Total**	19	76	17	68	16	68

### Appendix C. Activity Differences Among Three Categories of Race/Ethnicity and Among Family Affluence

In Figures S.1 and S.2, we show the observed *F*-statistic results and related permutation critical values for a relationship between activity counts and race/ethnicity, and between activity counts and family affluence, respectively.
